# The role of T‐type calcium channels in elderly human vascular function: A pilot randomized controlled trial

**DOI:** 10.1113/EP091645

**Published:** 2024-03-06

**Authors:** Ulrik Winning Iepsen, Andreas R. Hjortdal, Anne D. Thuesen, Stine H. Finsen, Pernille B. L. Hansen, Stefan P. Mortensen

**Affiliations:** ^1^ Center for Physical Activity Research Copenhagen University Hospital – Rigshospitalet Copenhagen Denmark; ^2^ Department of Anaesthesia and Intensive Care Copenhagen University Hospital – Hvidovre Hospital Copenhagen Denmark; ^3^ Department of Cardiovascular and Renal Research University of Southern Denmark Odense Denmark

**Keywords:** acetylcholine receptor, blood vessels, cardiovascular, endothelium, hypertension

## Abstract

Endothelial dysfunction develops with age and may precede cardiovascular disease. Animal data suggest that T‐type calcium channels play an important role in endothelial function, but data from humans are lacking. This study included 15 healthy, sedentary, elderly males for a double blinded, randomized controlled trial. For 8 weeks, they were given 40 mg/day of either efonidipine (L‐ and T‐type calcium channel blocker (CCB)) or nifedipine (L‐type CCB). Vascular function was evaluated by graded femoral arterial infusions of acetylcholine (ACh; endothelium‐dependent vasodilator) and sodium nitroprusside (endothelium‐independent vasodilator) both with and without co‐infusion of *N*‐acetylcysteine (NAC; antioxidant). We measured leg blood flow and mean arterial pressure and calculated leg vascular conductance to evaluate the leg vascular responses. Despite no significant change in blood pressure in either group, we observed higher leg blood flow responses (Δ 0.43 ± 0.45 l/min, *P *= 0.006) and leg vascular conductance (Δ 5.38 ± 5.67 ml/min/mmHg, *P *= 0.005) to intra‐arterial ACh after efonidipine, whereas there was no change in the nifedipine group, and no differences between groups. We found no upregulation of endothelial nitric oxide synthase in vastus lateralis muscle biopsies within or between groups. Smooth muscle cell responsiveness was unaltered by efonidipine or nifedipine. Intravenous co‐infusion of NAC did not affect endothelium‐dependent vasodilatation in either of the CCB groups. These results suggest that 8 weeks’ inhibition of T‐ and L‐type calcium channels augments endothelium‐dependent vasodilatory function in healthy elderly males. Further studies are required to elucidate if T‐type calcium channel inhibition can counteract endothelial dysfunction.

## Abstract (highlights)

- **What is the central question of this study?**
  Does T‐type calcium channel inhibition prevent age‐related endothelium dysfunction in humans?
- **What is the main finding and its importance?**
  Eight weeks of L+T‐type calcium channel blockade (CCB) increased endothelium‐dependent vasodilatation in the leg of otherwise healthy elderly males, while 8 weeks of L‐type CCB only did not. Thus, T‐type calcium channels may be involved in endothelium dysfunction.

## 1 INTRODUCTION

The endothelium plays a pivotal role in the regulation of vascular tone (Mortensen & Saltin, 2014; Widlansky et al., 2003). Endothelial dysfunction precedes the development of atherosclerosis and is associated with increased risk of morbidity in established cardiovascular disease, including hypertension and coronary artery disease (Heitzer et al., 2001; Yeboah et al., 2007). The loss of vasodilatory function is a hallmark of endothelium dysfunction, where the nitric oxide (NO) synthesis and release may be impaired, and thus, endothelium‐dependent vasodilatation has been reported to decline with age (Celermajer et al., 1994a; Deng et al., 1999; Taddei et al., 1995; Trinity et al., 2016).

Pharmacological inhibition of voltage‐gated calcium channels (Ca_v_) affects cardiovascular regulation by relaxation of vascular smooth muscle of resistance arterioles, NO release from endothelial cells, and reduction of myocardial contractility and chronotropy (Zamponi et al., 2015). Calcium channel blockers (CCB) have been suggested to augment endothelium‐dependent vasodilatation in the forearm, assessed by flow‐mediated dilatation (Radenković et al., 2019), and although mostly L‐type calcium channels are targeted clinically, emerging evidence suggests that T‐type calcium channels (Ca_v_3.1, Ca_v_3.2) are involved in vascular vasodilatory function (Hansen, 2015). Interestingly, animal studies show that Ca_v_3.1 is co‐localized with endothelial NO synthase (eNOS), and thereby increases bioavailability of NO and NO‐mediated vasodilatation (Svenningsen et al., 2014). T‐type calcium channel‐deficient mice (Ca_v_3.1^−/−^, Ca_v_3.2^−/−^) develop less age‐dependent endothelial dysfunction (Thuesen et al., 2018), suggesting that T‐type channels play an important role in age‐induced endothelial dysfunction even though the ageing T‐type deficient mice remained normotensive. Thus, the vascular effects of T‐type CCB could be protective against the development of cardiovascular disease in an aged population, but evidence is based on animal models or patients with established cardiovascular disease, and physiological mechanisms in elderly, healthy humans are not well established.

This randomized double‐blinded controlled trial investigated the effects of 8 weeks of efonidipine (combined L‐and T‐type CCB) versus nifedipine (L‐type CCB) in 15 elderly, healthy males (≥60 years). The primary outcome was vascular function evaluated by leg blood flow (LBF) and mean arterial pressure (MAP) as well as leg vascular conductance (LVC) in response to arterial infusions of (1) acetylcholine (ACh) to test the endothelium‐dependent vasodilatory function; (2) sodium nitroprusside (SNP) to test the endothelium‐independent vasodilatory function; and (3) ACh and SNP with co‐infusion of *N*‐acetylcysteine (NAC). The secondary outcome was leg muscle content of eNOS evaluated by western blotting. Our hypothesis was that endothelium‐dependent vasodilatation is improved by efonidipine through higher NO availability and lower oxidative stress compared with nifedipine.

## 2 METHODS

### 2.1 Ethical approval

The project was approved by the Ethics Committee of Copenhagen and Region of Southern Denmark (H‐15005648) and registered at ClinicalTrials.gov (NCT02885558). The project was conducted in accordance with the *Declaration of Helsinki*. All participants were informed both verbally and in writing before informed consent was obtained.

### 2.2 Participants

This was a double‐blinded randomized controlled trial. The researchers that enrolled participants and analysed data were blinded. Fifteen healthy elderly male individuals were included in the per‐protocol analysis and one participant withdrew consent.

Participants with hypertension (blood pressure > 140/90 mmHg), body mass index > 35 kg/m^2^, who performed exercise more than 2 h/week or were smokers were excluded. All the participants went through a pre‐experimental day consisting of a resting 12‐lead electrocardiogram (MAC800, GE Medical systems, Milwaukee, WI, USA), blood pressure measuring (OMRON M3, Comfort, Kyoto, Japan) and a fasting blood screening (haematology, lipids and kidney function). None of the participants were diagnosed with cardiovascular disease or demonstrated evidence of renal disease defined as estimated glomerular filtration rate <60 ml/min/1.73 m^2^ based on plasma creatinine levels, sex and age.

### 2.3 Experimental design

**FIGURE 1:**
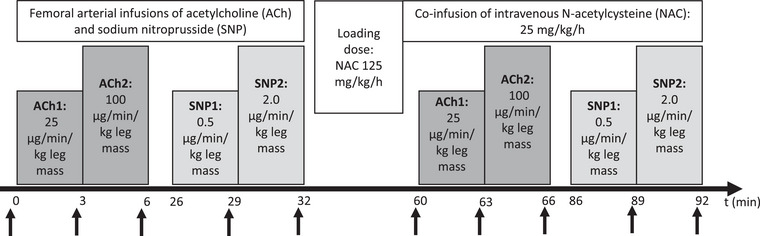
Study protocol. Catheters were placed in the femoral artery and vein, and a muscle biopsy was obtained from the contralateral vastus lateralis. The experiment began with supine rest for 20 min (baseline) followed by femoral arterial infusion of acetylcholine (ACh) at two infusion rates (ACh 1: 25 µg/min/kg leg mass, ACh2: 100 µg/min/kg leg mass) followed by 20 min of supine rest. Likewise, sodium nitroprusside (SNP) was infused at two rates (SNP1: 0.5 µg/min/kg leg mass, SNP2: 2.0 µg/min/kg leg mass). Then an intravenous *N*‐acetylcysteine (NAC) loading dose for 20 min (125 mg/min/kg total body mass) was initiated followed by maintenance dose (25 mg/min/kg total body mass), which was continued for the rest of the experiment and during the same doses of ACh and SNP, as before NAC. Leg blood flow and intravascular blood pressure were measured at the end of each infusion trial marked by black arrows.

The participants completed an experimental day before (pre) and after (post), 8 weeks of treatment with efonidipine or nifedipine, starting at 20 mg/day for the first 2 weeks and then increased to 40 mg/day. Twenty‐four hours prior to each experimental day, all participants refrained from caffeine, alcohol and exercise. On each experimental day, the subjects ingested a light standardized breakfast at 06.00 h and arrived at the laboratory at 08.30 h. Under aseptic conditions and local anaesthesia (xylocaine, 10 mg/ml, AstraZeneca, Mölndal, Sweden), three catheters (18GA, Arrow International, Reading, PA, USA) were placed by ultrasound guidance, at a level just proximal for the bifurcator of the common femoral artery, using the Seldinger technique. Two catheters were placed in the experimental leg, respectively in the femoral artery (pharmacological infusions) and vein (blood samples), and one catheter was placed in the femoral artery of the non‐experimental leg (blood sampling and blood pressure measurements). After local anaesthesia of the skin and muscle fascia, the percutaneous needle biopsy technique with suction was used to collect the biopsy from the middle portion of m. vastus lateralis of the left leg and immediately frozen in liquid nitrogen. After 20 min of rest, the participants completed the following trial (Figure 1): (1) ACh infusion (Miochol‐E, Bausch + Lomb, Berlin, Germany): two stepwise 2.5 min infusions at 25 (ACh1) and 100 (ACh2) µg/min/kg leg volume; (2) SNP (Nitropress, Hospira, Lake Forrest, IL, USA): two stepwise 2.5 min infusions at 0.5 (SNP1) and 2 (SNP2) µg/min/kg leg volume; (3) NAC infusion (Amgros I/S, Copenhagen, Denmark): 20 min at 125 mg/h/kg total body mass (loading dose) and subsequently at 25 mg/h/kg total body mass (maintenance dose), for the rest of the experimental protocol to potentiate NO bioavailability; and (4) after 50 min of NAC infusion, ACh was repeated. Each trial was separated by 20–30 min of rest.

### 2.4 Measurements and calculations

Anthropometric measurements were used for calculation of leg volume. Femoral arterial blood flow (leg blood flow; LBF) was measured by Doppler ultrasound (Logic E9, GE Healthcare, Milwaukee, WI, USA), as previously described (Mortensen et al. 2012), the analyser being blinded towards group assignment. Mean arterial pressure (MAP) and heart rate (HR) were monitored with transducers positioned at the level of the heart (pressure monitoring kit, ref. T450217A; Edwards Lifesciences, Irvine, CA, USA). Recordings were made via a data acquisition system (PowerLab 16/30, ADInstruments, Bella Vista, NSW, Australia), for later software analysis (Labchart 8, ADInstruments). MAP (mmHg) and HR (beats/min) were calculated over 8–16 cycles from the area under the arterial pressure curve. Leg vascular conductance was calculated as LBF/MAP. Arterial and venous blood samples were drawn simultaneously before and during the individual infusion trials (2 min), for determination of O_2_ variables. Blood samples were immediately (<5 min) analysed for blood gas variables (ABL835, Radiometer, Copenhagen, Denmark). Arterial and venous EDTA–blood were obtained simultaneously and centrifuged <15 min (4°C) at 3500 *g* for 10 min. Plasma aliquots were frozen at −80°C awaiting analysis. The stable metabolites nitrite and nitrate (NOx) in plasma from the femoral artery and vein were analysed using fluorometric assay kit (Cayman Chemical Co., Ann Arbor, MI, USA) following the manufacturer's instructions.

Blood pressure (BP) was measured using an automated, validated BP‐device (M6, Omron, Kyoto, Japan), by the participant at home 3 × 3 times a day 3 days before, during the first and last week of treatment. In the 6 weeks in between, BP was measured 3 × 3 times a day every second day. Participants were instructed verbally and in writing how to measure BP and given a schedule to fill in values, which was reported back to the investigator. The average BP over the 3 days before, and the last 3 days of the CCB treatment were used as BP before and after the intervention.

### 2.5 Western blotting

Western immunoblotting (WB) was performed on muscle biopsies from pre‐ and post intervention with CCB treatment. Protein was extracted from the muscle biopsy with RIPA Lysis Buffer (10×; Millipore Corp, Billerica, MA, USA) and cOmplete, Mini Protease Inhibitor Cocktail (Roche, Mannheim, Germany) was added to the lysis buffer. Concentration of the lysate was determined by a Pierce BCA Protein Assay Kit (Thermo Fisher Scientific, Waltham, MA, USA) and measured on a VERSAmax Tuneable Microplate Reader (Molecular Devices, San Jose, CA, USA) to ensure equal protein concentrations between samples. Protein lysate was mixed with NuPAGE Sample Reducing Agent (10×; Thermo Fisher Scientific), NuPAGE LDS Sample Buffer (4×; Thermo Fisher Scientific) and heat denatured. Samples were loaded onto the gels with both samples from one individual (before and after 8 weeks of CCB intervention) side by side on the same gel and were run on a Mini‐PROTEAN TGX Precast Gels 4−15% (Bio‐Rad Laboratories, Hercules, CA, USA) at 200 V, 70 mA for ∼35 min, and subsequently blotted onto a Midi format polyvinylidene difluoride 0.2 µm Trans‐Blot Turbo Transfer membrane (Bio‐Rad Laboratories). Membranes were blocked in 5% milk in Tris‐buffered saline with 0.1% Tween 20 (TBST) and then presented with the following primary antibodies: purified mouse anti‐eNOS/NOS Type III (BD Transduction Laboratories, Franklin Lakes, NJ, USA) and rabbit polyclonal to glyceraldehyde 3‐phosphate dehydrogenase (GAPDH) loading control (Abcam, Cambridge, UK) or β‐actin (Abcam, Cambridge, UK). For secondary antibodies, polyclonal goat anti‐mouse immunoglobulins/horseradish peroxidase (HRP) (Dako, Agilent Technologies Denmark Aps, Glostrup, Denmark) and polyclonal goat anti‐rabbit immunoglobulins/HRP (Dako, Agilent Technologies) were used. All antibodies were diluted in TBST. Blots were developed using Western Lighting ECL Pro (Perkin Elmer, Inc., Waltham, MA, USA). For anti‐eNOS/NOS Type III, the enhancer SuperSignal West Femto Maximum Sensitivity Substrate (Thermo Fisher Scientific) was supplemented (1:4) to the ECL mix. For imaging, Molecular Imager Chemidoc XRS+ (Bio‐Rad Laboratories) was used. The semi‐quantification was done in Image Lab (Bio‐Rad Laboratories) relative to GAPDH or β‐actin.

### 2.6 Statistical analysis

The mean changes from baseline (delta values, Δ) in haemodynamic responses to vasoactive infusions were calculated and a two‐way repeated measures analysis of variance (ANOVA) was used to detect differences from pre‐CCB to post‐CCB within groups. The mean change from pre‐CCB to post‐CCB was used to compare haemodynamics at rest and during vasoactive infusions, and differences between the two groups were detected using a 2 × 2 mixed model ANOVA. We did Bonferroni adjustment for multiple comparisons. We did not calculate sample size a priori, as this was a first‐in‐human investigation. All statistical analyses were conducted using IBM SPSS Statistics (v. 28.0.0.0, IBM Corp., Armonk, NY, USA). *P*‐values < 0.05 were considered significant. All data are presented as means with standard deviations (SD) unless otherwise stated.

## 3 RESULTS

Characteristics, including average resting BP values measured at home (three times per day on three consecutive days), of participants in the efonidipine and nifedipine groups are presented in Table 1. There was no significant change in systolic BP (efonidipine −5 ± 10 mmHg, *P* = 0.330 vs. nifedipine −10 ± 10 mmHg, *P* = 0.230) or diastolic BP (efonidipine −4 ± 5 mmHg, *P *= 0.100 vs. nifedipine −3 ± 11, *P *= 0.570) with CCB treatment in either group, with no difference between groups (systolic BP, *P *= 0.520; diastolic BP, *P *= 0.830).

**TABLE 1:** Baseline characteristics.

	Nifedipine (*n* = 7)		Efonidipine (*n* = 7)	
	Baseline	8 weeks follow‐up	Baseline	8 weeks follow‐up
Sex (male/female)	7/0		7/0	
Age (years)	65 (4)		66 (2)	
BMI (kg/m^2^)	26.3 (3.8)		29.0 (1.7)	
Medication (*n* (%))				
Statins	1 (14)		2 (29)	
ASA	0 (0)		2 (29)	
Dipyridamole	0 (0)		1 (14)	
Blood pressure at home (mmHg)				
Systolic BP	133 (15)	123 (16)	126 (9)	121 (10)
Diastolic BP	82 (10)	79 (10)	80 (5)	76 (4)
Heart rate (beats/min)	58 (11)	61 (16)	65 (10)	64 (6)
Blood tests				
Cholesterol (mmol/l)	5.1 (1.2)	4.7 (0.7)	5.0 (0.7)	4.4 (1.2)
LDL (mmol/l)	3.2 (1.4)	3.0 (0.6)	3.1 (1.0)	2.6 (1.1)
HDL (mmol/l)	1.1 (0.2)	1.2 (0.2)	1.2 (0.2)	1.2 (0.2)
Triglycerides (mmol/l)	1.8 (1.0)	1.1 (0.4)	1.4 (0.3)	1.3 (0.2)
Creatinine (mmol/l)	88 (11)	82.2 (11.2)	85 (5)	82 (6)
eGFR (ml/min/1.73^2^)	80 (12)	80 (12)	81 (5)	85 (6)
K^+^ (mmol/l)	4.2 (0.4)	4.0 (0.2)	4.3 (0.5)	4.0 (0.2)
Femoral venous NO concentrations				
Baseline rest (µmol/l)	10.2 (5.4)	9.2 (2.1)	7.5 (2.5)	9.9 (3.9)
NAC co‐infusion (µmol/l)	10.5 (4.2)	9.3 (2.3)	8.3 (2.9)	9.7 (3.3)
Femoral arterial NO concentrations				
Baseline rest (µmol/l)	10.5 (4.9)	9.0 (2.4)	7.5 (2.8)	9.7 (4.3)
NAC co‐infusion (µmol/l)	10.6 (4.2)	9.2 (2.3)	8.3 (2.8)	10.2 (3.2)

*Note*: Data are means with (standard deviations) or count (%). NO concentrations are based on *n* = 5 (nifedipine) versus *n* = 6 (efonidipine). Abbreviations: ASA, acetylsalicylic acid; BMI, body mass index; BP, blood pressure; eGFR, estimated glomerular filtration rate; HDL, high density lipoprotein; LDL, low density lipoprotein; NAC, *N*‐acetylcysteine; NO, nitric oxide.

### 3.1 Leg blood flow

**FIGURE 2:**
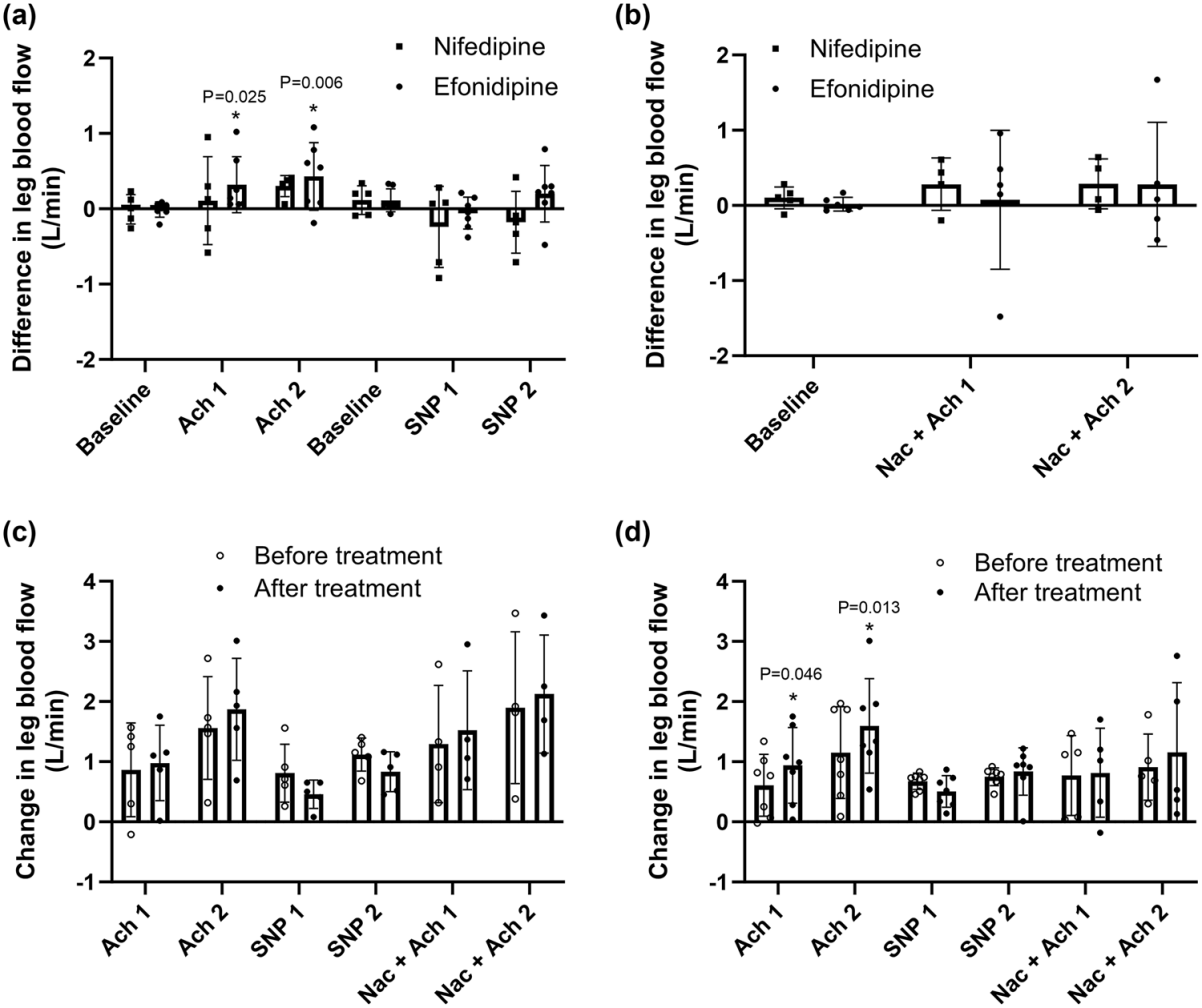
Leg blood flow. (a) Nifedipine versus efonidipine by the difference in leg blood flow between pre‐trial and post‐trial at baseline and during femoral arterial infusions of acetylcholine 25 µg/min/kg leg mass (ACh1), acetylcholine 100 µg/min/kg leg mass (ACh2), sodium nitroprusside 0.5 µg/min/kg leg mass (SNP1) and sodium nitroprusside 2 µg/min/kg leg mass (SNP2). (b) Nifedipine versus efonidipine by the differences in leg blood flow between pre‐trial and post‐trial during baseline and during ACh1 and ACh2 with co‐infusion of *N*‐acetylcysteine 25 mg/min/kg total body mass (NAC). (c) The leg blood flow responses from baseline within nifedipine group at pre‐ and post‐treatment. (d) The leg blood flow responses from baseline within efonidipine group at pre‐ and post‐treatment. Bars represent means, and whiskers represent SD. *n* = 5 (nifedipine) versus *n* = 7 (efonidipine).

During ACh2, LBF in the efonidipine group was 1.38 ± 0.82 pre‐CCB versus 1.82 ± 0.81 l/min post‐CCB, while LBF in the nifedipine group was 1.82 ± 0.88 pre‐CCB versus 2.12 ± 0.88 l/min post‐CCB, with no significant differences between groups (Figure 2). In the efonidipine group, LBF was higher during ACh1 (*P *= 0.025) and ACh2 (*P *= 0.006) after CCB treatment, whereas no significant changes were observed in the nifedipine group. There were no differences between groups in LBF responses to SNP before and after efonidipine or nifedipine and we found no effect of CBB on the blood flow response to SNP. We found no differences within or between groups in LBF responses to ACh with co‐infusion of NAC.

### 3.2 Mean arterial pressure

**FIGURE 3:**
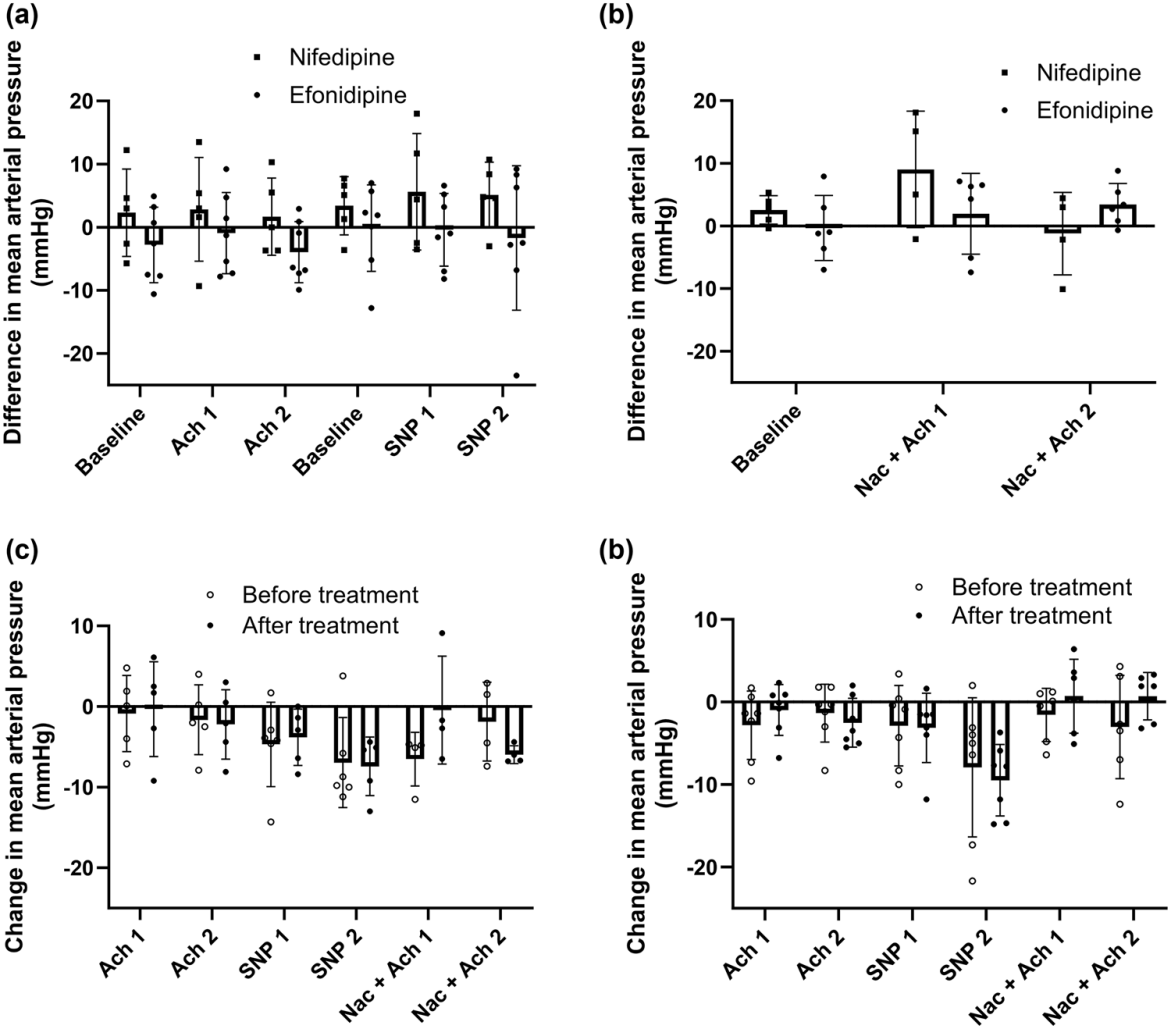
Mean arterial pressure. (a) Nifedipine versus efonidipine by the difference in mean arterial pressure (MAP) between pre‐trial and post‐trial at baseline and during femoral arterial infusions of acetylcholine 25 µg/min/kg leg mass (ACh1), acetylcholine 100 µg/min/kg leg mass (ACh2), sodium nitroprusside 0.5 µg/min/kg leg mass (SNP1) and sodium nitroprusside 2 µg/min/kg leg mass (SNP2). (b) Nifedipine versus efonidipine by the differences in MAP between pre‐trial and post‐trial during baseline and during ACh1 and ACh2 with co‐infusion of *N*‐acetylcysteine 25 mg/min/kg total body mass (NAC). (c) The MAP responses from baseline within nifedipine group at pre‐ and post‐treatment. (d) The MAP responses from baseline within efonidipine group at pre‐ and post‐treatment. Bars represent means, and whiskers represent SD. *n* = 5 (nifedipine) versus *n* = 7 (efonidipine).

MAP in the efonidipine group was 97 ± 12 pre‐CCB versus 93 ± 11 mmHg post‐CCB during ACh2. In the nifedipine group, the pre‐CCB MAP was 93 ± 15 versus 97 ± 8 mmHg post‐CCB, with no differences between groups (Figure 3). We found no between‐ or within‐group differences in MAP responses to SNP. Likewise, there was no change from pre‐ to post‐MAP during SNP infusion in either of the groups. There were no differences between or within the groups in MAP responses to ACh with co‐infusion of NAC. The systolic or diastolic blood pressure responses were similarly unaffected (Figures A1 and A2—see Appendix).

### 3.3 Leg vascular conductance

**FIGURE 4:**
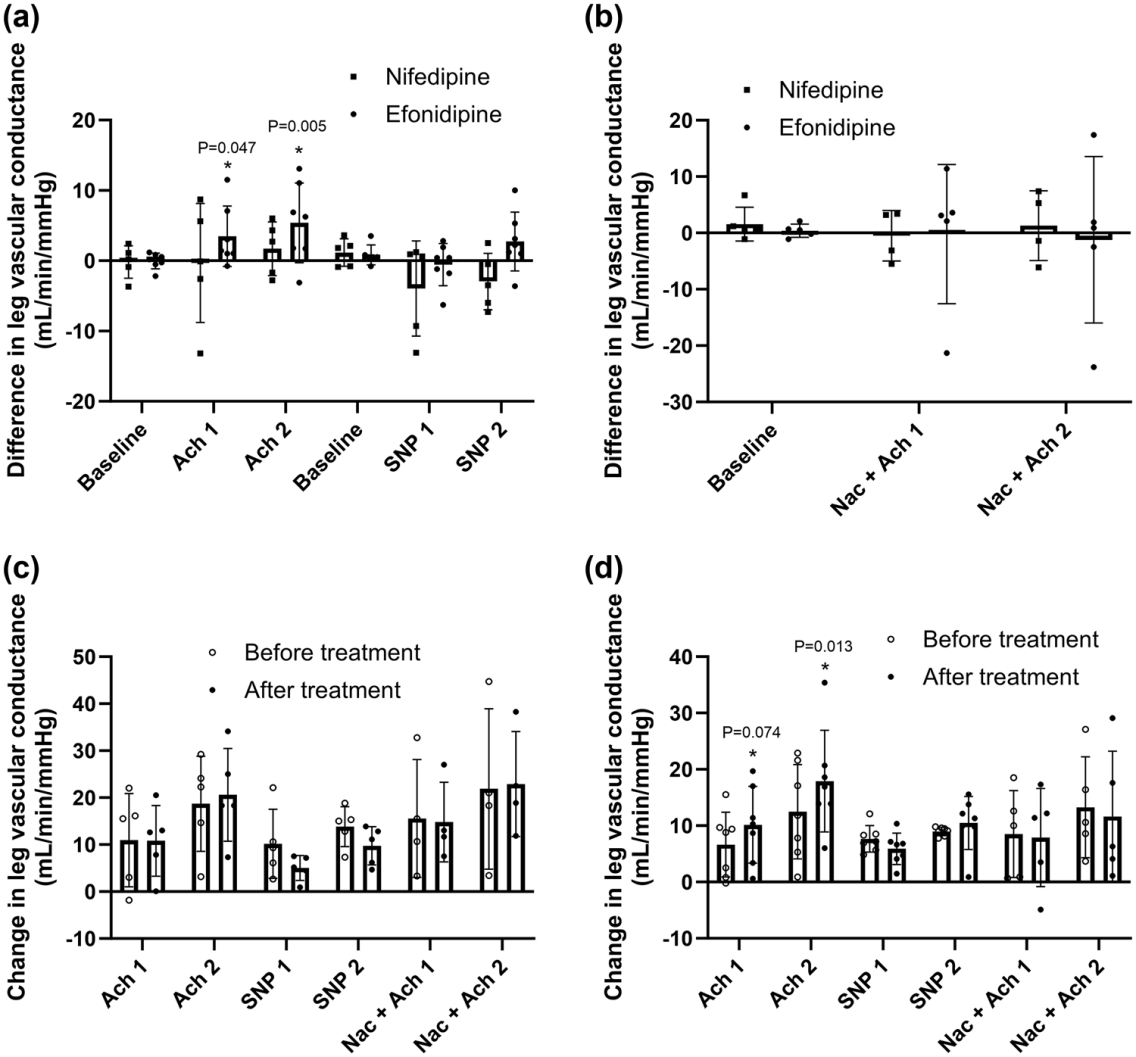
Leg vascular conductance. (a) Nifedipine versus efonidipine by the difference in leg vascular conductance (LVC) between pre‐trial and post‐trial at baseline and during femoral arterial infusions of acetylcholine 25 µg/min/kg leg mass (ACh1), acetylcholine 100 µg/min/kg leg mass (ACh2), sodium nitroprusside 0.5 µg/min/kg leg mass (SNP1) and sodium nitroprusside 2 µg/min/kg leg mass (SNP2). (b) Nifedipine versus efonidipine by the differences in LVC between pre‐trial and post‐trial during baseline and during ACh1 and ACh2 with co‐infusion of *N*‐acetylcysteine 25 mg/min/kg total body mass (NAC). (c) The LVC responses from baseline within nifedipine group at pre‐ and post‐treatment. (d) The LVC responses from baseline within efonidipine group at pre‐ and post‐treatment. Bars represent means, and whiskers represent SD. *n* = 5 (nifedipine) versus *n* = 7 (efonidipine).

There were no differences between groups in LVC responses to ACh infusion before or after CCB treatment. In the efonidipine group, LVC was higher in response to ACh1 (*P *= 0.047) and ACh2 (*P *= 0.005) after CCB treatment, whereas there was no effect of CCB in the nifedipine group (Figure 4). There was no difference between groups in their LVC responses to SNP. Both groups increased LVC during SNP infusion both before and after CCB. We found no differences between groups in LVC responses to co‐infusion of NAC and ACh, but dose‐dependent increases in LVC (Δ 4.1 ± 1.3 ml/min/mmHg, *P *= 0.032) and (Δ 5.7 ± 1.8 ml/min/mmHg, *P *= 0.049) were seen in the efonidipine and nifedipine group, respectively.

### 3.4 NO bioavailability

**FIGURE 5:**
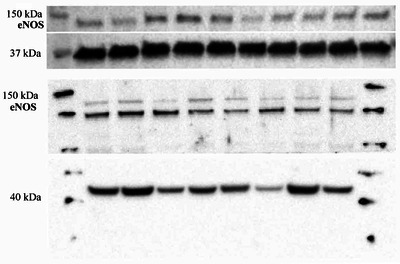
Muscle content of endothelial nitric oxide synthase (eNOS). There was no difference within or between groups in muscle content of eNOS, *n* = 3 (nifedipine) versus *n* = 5 (efonidipine).

We found no change in the plasma NOx concentrations in the femoral artery or vein with CBB in either group, with no differences between groups (Table 1). Likewise, there were no differences within or between groups in muscle content of eNOS (Figure 5).

## 4 DISCUSSION

We studied the effects of 8 weeks combined L‐ and T‐type CCB (efonidipine) versus L‐type CCB alone (nifedipine) on endothelial function in older otherwise healthy males in a randomized design. After 8 weeks, we observed higher LBF responses to intra‐arterial ACh within the efonidipine group, suggesting an augmented endothelium‐dependent vasodilatation and no change in the nifedipine group, with no differences between groups. We found no differences in metabolites of NO formation in plasma or upregulation of eNOS in skeletal muscle within or between groups. Likewise, intravenous infusion of an antioxidant (NAC) did not affect endothelium‐dependent or ‐independent vasodilatation in either of the CCB groups.

### 4.1 Endothelial function in health and disease

The effect of T‐type CCB blockade on endothelial dysfunction has not to our knowledge been studied previously in older healthy humans. Endothelium‐dependent vasodilatation may already begin to decline at age 40 in otherwise healthy men, while it is postponed in women until the menopause (Celermajer et al., 1994b; Gerhard et al., 1996). As our participants were >60‐year‐old men, we considered that they had some degree of age‐related endothelial dysfunction, but importantly not yet manifest cardiovascular disease. This window of opportunity is crucial when trying to explore interventions to preserve vascular health in an ageing population before they develop cardiovascular diseases (Christensen et al., 2009). Our findings that inhibition of both L‐ and T‐type calcium channels increase LBF responses during intra‐arterial ACh by approximately 33% indicate that there is a potential for improving age‐related reductions in endothelium‐dependent vasodilatation. Sufficient blood flow to supply leg muscles with oxygen is required to maintain ambulatory function, but ageing impairs resting LBF by approximately 26% in healthy 63‐year‐old men compared with 35‐year‐old younger controls (Dinenno et al., 1999). The insufficient vasodilatory function associated with ageing may lead to a vicious circle of lower physical performance, deconditioning and further vascular dysfunction, increasing the risk of lifestyle‐related cardiovascular disorders. While the participants in the present study were physically inactive, evidence shows that lifelong adherence to physical activity largely preserves endothelial function in older, healthy individuals, and they maintain similar blood supply to the working leg muscles compared with young individuals (Mortensen et al., 2012). Thus, one of the key elements of healthy ageing relies on preserving vascular function to maintain a physically active lifestyle.

Endothelial dysfunction develops with age (Celermajer et al., 1994b) and is considered to precede arthrosclerosis followed by cardiovascular disease. The main pathophysiological mechanism is thought to be reduced NO bioavailability caused by accumulated oxidative stress and inflammation with ageing, which impairs the vasodilatory and anti‐coagulant functions of the healthy endothelium (Seals et al., 2014).

### 4.2 NAC and NO bioavailability

Previous investigations in humans have suggested that efonidipine improves endothelial function, but evidence is only based on individuals with pre‐existing cardiovascular disease, such as hypertension or diabetes (Kwang et al., 2007; Oshima et al., 2005; Sasaki et al., 2009), and generally CCB may improve endothelial function in this population (Radenković et al., 2019). Our understanding of the mechanisms of the proposed vascular effects of efonidipine is related to its T‐type CCB but limited to animal studies showing Ca_v_3.1 involvement in NO production and thereby improved endothelial vasodilatory function (Hansen, 2015; Svenningsen et al., 2014). Therefore, a secondary aim of the present study was to elucidate vasodilatory mechanisms, with focus on change in the NO bioavailability, after chronic T‐type CCB in healthy elderly humans. However, our data did not suggest that muscle content of eNOS or metabolites of NO in plasma are upregulated after efonidipine treatment. Likewise, introducing a reactive oxygen species scavenger by co‐infusion of NAC systemically and thereby theoretically increasing NO availability did not alter endothelium‐dependent vasodilatory function in the efonidipine or nifedipine group. We did not observe changes in the vasodilatory capacity in response to intra‐arterial infusions of SNP, an NO donor, after efonidipine or nifedipine. These observations might be explained by the admittedly low number of subjects or maybe more likely by the remarkable redundancy of the human vasculature (Clifford & Hellsten, 2004). Numerous other endogenous vasodilatory agents have been proposed to be of importance, including ATP, adenosine, potassium, H^+^ and endothelium‐derived hyperpolarizing factor, but regulation of blood flow by the muscle vasculature has not been attributed to a single vasodilator (Clifford & Hellsten, 2004). Therefore, we suspect that even if upregulation of NO actually did occur, other vasodilators may just have been downregulated and our results more likely indicate a global improvement in endothelial function, rather than specifically targeting NO availability.

### 4.3 Blood pressure regulation

Only normotensive participants without cardiovascular indications for CCB were studied, and this is likely to explain why we did not observe a BP lowering effect of CCB after 8 weeks. The BP was not the primary outcome of interest, as we have previously reported that endothelium dysfunction developed within wild‐type mice, but not in the T‐type knock‐out model, despite the ageing mice remaining normotensive (Thuesen et al., 2018). Herein, we studied if we could mitigate endothelium function in healthy normotensive elderly by blocking T‐type calcium channels independently of BP, with the rationale of prevention against development of cardiovascular disease including hypertension by blocking T‐type calcium channels. As blood pressure remained unchanged in this population, we cannot exclude that a more aggressive lowering of the BP by increasing dose or duration would have affected endothelium function to a greater extent. Independent of whether they are T‐ or L‐type, CCBs have been suggested to increase global sympathetic nervous activity through a baroreflex response to lowering of BP (Lindqvist et al., 2007). Importantly, the evidence of this notion is based on individuals with hypertension, who already have higher sympathetic outflow at baseline (Delaney et al., 2010; Kobetic et al., 2022). We did not assess sympathetic nerve activity, but if sympathetic outflow were lowered by efonidipine and not by nifedipine, this could explain the higher vascular conductance during ACh we observed in the efonidipine group (Fairfax et al., 2013). Although, we cannot rule out a difference in sympathetic activation, our results do not indicate a global difference in sympathoexcitation between groups, as the haemodynamic responses to SNP (endothelium‐independent vasodilatation) were similar.

### 4.4 Limitations

We only included healthy male subjects because evidence indicates that the age‐related decline in vascular function may develop in a more rapid non‐linear fashion after menopause in women (Celermajer et al., 1994a; Tamariz‐Ellemann et al., 2023), which was expected to confound comparisons with males of similar age. Thus, as we did not stratify for endothelium function upon inclusion for practical reasons (invasiveness of the trial) to match endothelium function between sexes; we only investigated male subjects. A general challenge for this kind of study is sample size, which is relatively small because of the invasive nature of these experiments. Thus, some differences within and between the groups may not have been detected due to lack of power. Also, there is a wide variation of the dependent variable of our primary outcome, leg blood flow. This again means that larger sample sizes may be needed to detect possible differences. In this study, trial participants were treated with CCB for 8 weeks, whereas in previous studies of patients with cardiovascular disease, treatment has been evaluated with longer follow‐up (Oshima et al., 2005; Seals et al., 2014). Thus, the full potential of the CCB may not yet be seen. Two participants in the efonidipine group were taking medication, statins, acetylsalicylic acid and dipyridamole, all of which may have influence on the endothelial function (Husain et al., 1998; Kim & Liao, 2008; Wolfrum et al., 2003).

### 4.5 Conclusion

These results suggest that 8 weeks’ inhibition of T‐ and L‐type calcium channels augments endothelium‐dependent vasodilatation in healthy elderly males. Further studies are required to elucidate if T‐type calcium channels play a role in the development of endothelial dysfunction with ageing.

## AUTHOR CONTRIBUTIONS

Conception and design: Pernille B. L. Hansen and Stefan P. Mortensen. Authors Ulrik Winning Iepsen, Anne D. Thuesen, Stine H. Finsen and Stefan P. Mortensen performed the experiments. Ulrik Winning Iepsen, Andreas R. Hjortdal, Anne D. Thuesen and Stefan P. Mortensen analysed and interpreted the data. Ulrik Winning Iepsen drafted the manuscript. All authors critically revised the manuscript for important intellectual content. All authors have read and approved the final version of this manuscript and agree to be accountable for all aspects of the work in ensuring that questions related to the accuracy or integrity of any part of the work are appropriately investigated and resolved. All persons designated as authors qualify for authorship, and all those who qualify for authorship are listed.

## CONFLICT OF INTEREST

The authors have no conflicts of interest.

5

**FIGURE A1:**
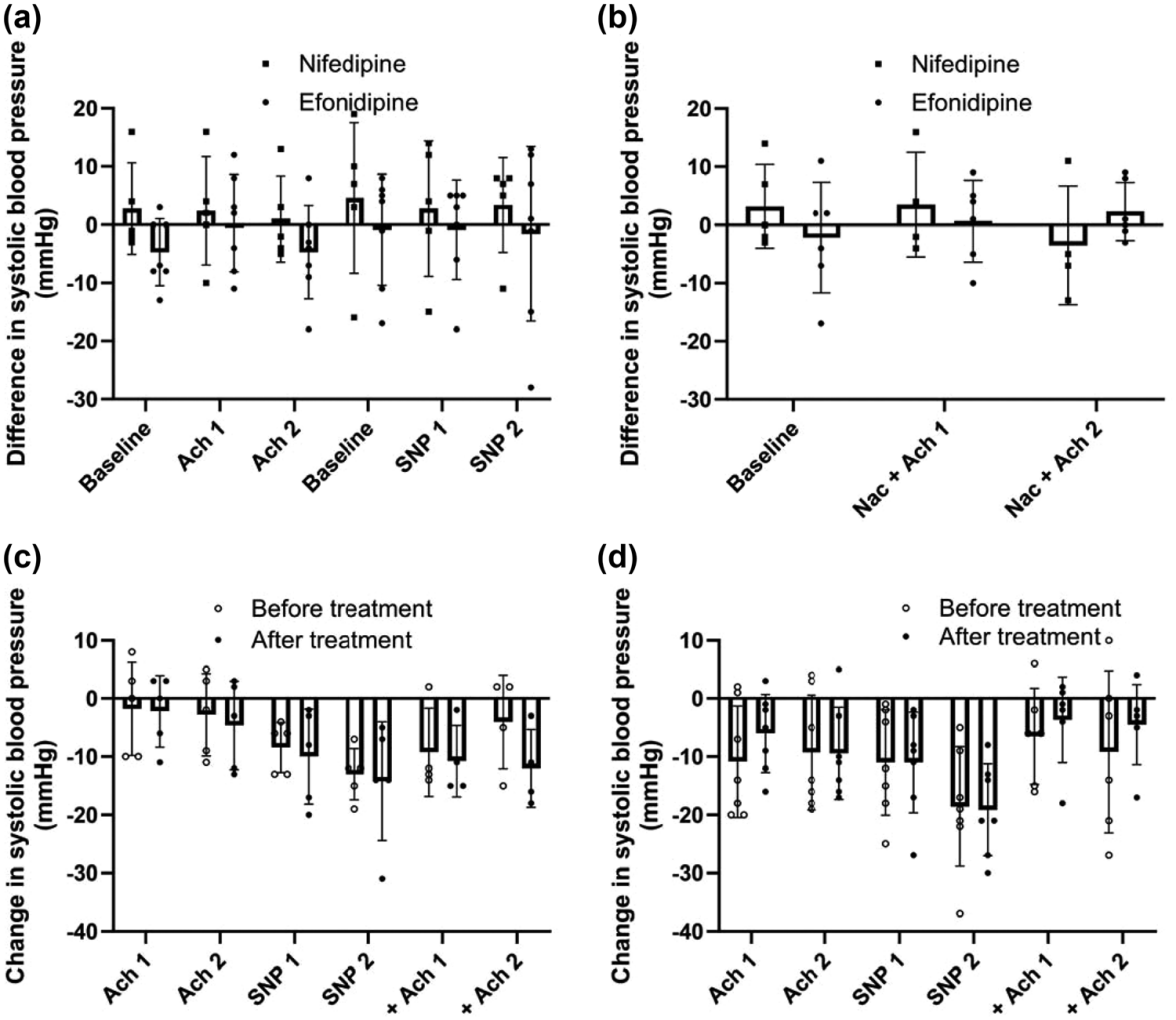
Systolic blood pressure. (a) Nifedipine versus efonidipine by the difference in systolic blood pressure between pre‐trial and post‐trial at baseline and during femoral arterial infusions of acetylcholine 25 µg/min/kg leg mass (ACh1), acetylcholine 100 µg/min/kg leg mass (ACh2), sodium nitroprusside 0.5 µg/min/kg leg mass (SNP1) and sodium nitroprusside 2 µg/min/kg leg mass (SNP2). (b) Nifedipine versus efonidipine by the differences in systolic blood pressure between pre‐trial and post‐trial during baseline and during ACh1 and ACh2 with co‐infusion of *N*‐acetylcysteine 25 mg/min/kg total body mass (NAC). (c) The systolic blood pressure responses from baseline within nifedipine group at pre‐ and post‐treatment. (d) The systolic blood pressure responses from baseline within efonidipine group at pre‐ and post‐treatment. Bars represent means, and whiskers represent SD. *n* = 5 (nifedipine) versus *n* = 7 (efonidipine).

**FIGURE A2:**
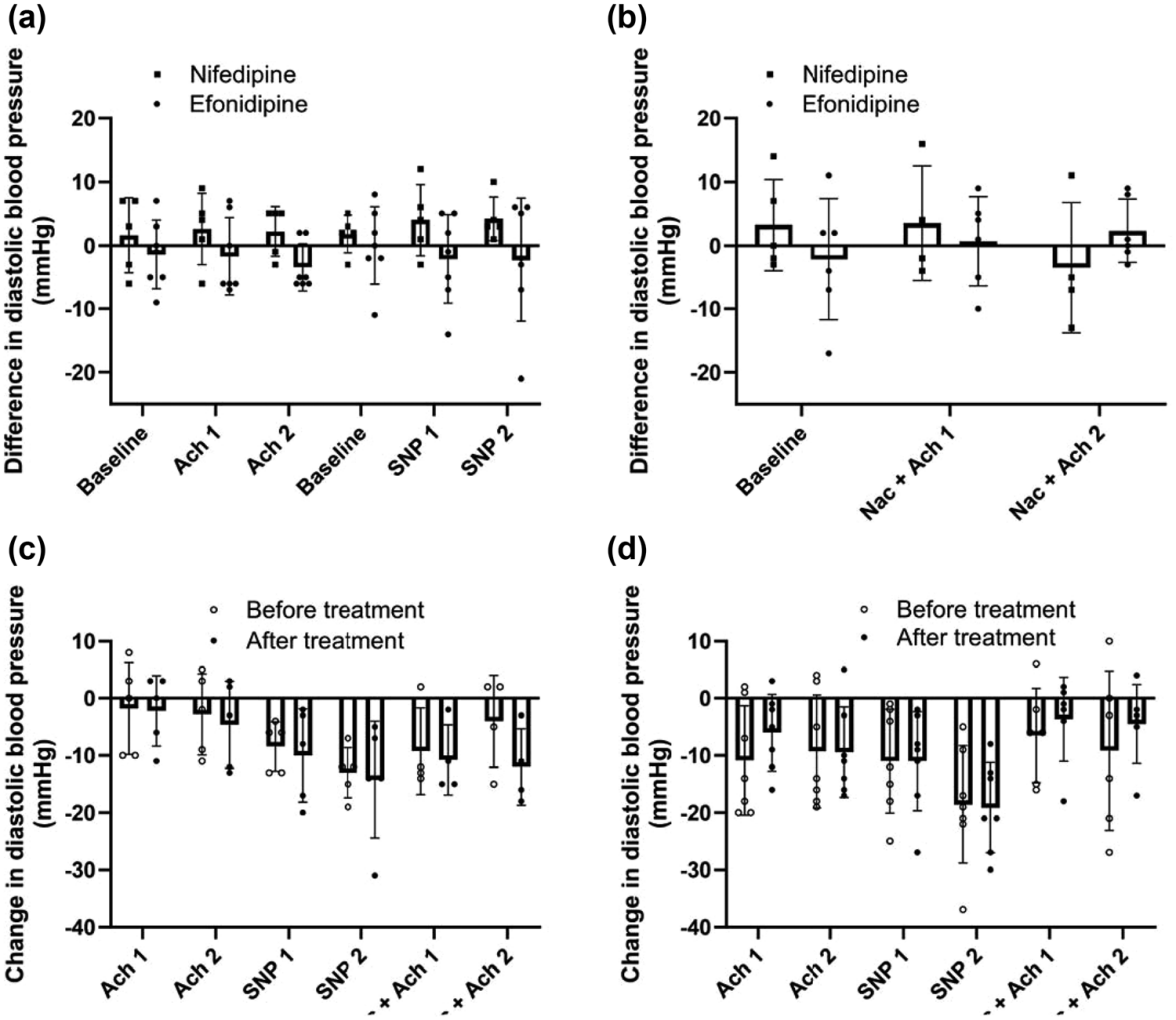
Diastolic blood pressure. (a) Nifedipine versus efonidipine by the difference in diastolic blood pressure between pre‐trial and post‐trial at baseline and during femoral arterial infusions of acetylcholine 25 µg/min/kg leg mass (ACh1), acetylcholine 100 µg/min/kg leg mass (ACh2), sodium nitroprusside 0.5 µg/min/kg leg mass (SNP1) and sodium nitroprusside 2 µg/min/kg leg mass (SNP2). (b) Nifedipine versus efonidipine by the differences in diastolic blood pressure between pre‐trial and post‐trial during baseline and during ACh1 and ACh2 with co‐infusion of *N*‐acetylcysteine 25 mg/min/kg total body mass (NAC). (c) The diastolic blood pressure responses from baseline within nifedipine group at pre‐ and post‐treatment. (d) The diastolic blood pressure responses from baseline within efonidipine group at pre‐ and post‐treatment. Bars represent means, and whiskers represent SD. *n* = 5 (nifedipine) versus *n* = 7 (efonidipine).

## Data Availability

The data that support the findings of the present study are available from the corresponding author upon reasonable request.

## 1

Figure A1. Systolic blood pressure.

Figure A2. Diastolic blood pressure.
